# Prognostic and therapeutic value of the Hippo pathway, RABL6A, and p53-MDM2 axes in sarcomas

**DOI:** 10.18632/oncotarget.27928

**Published:** 2021-04-13

**Authors:** Chandni Desai, Jon Thomason, Jordan L. Kohlmeyer, Anna C. Reisetter, Parmanand Ahirwar, Khadijeh Jahanseir, Mariah Leidinger, Georgina Ofori-Amanfo, Karen Fritchie, Sadanandan E. Velu, Patrick Breheny, Dawn E. Quelle, Munir R. Tanas

**Affiliations:** ^1^Department of Pathology, University of Iowa, Iowa City, IA, USA; ^2^Carver College of Medicine, University of Iowa, Iowa City, IA, USA; ^3^Department of Neuroscience and Pharmacology, University of Iowa, Iowa City, IA, USA; ^4^Molecular Medicine Graduate Program, University of Iowa, Iowa City, IA, USA; ^5^Department of Biostatistics, University of Iowa, Iowa City, IA, USA; ^6^Department of Chemistry, University of Alabama at Birmingham, Birmingham, AL, USA; ^7^Department of Laboratory Medicine and Pathology, Mayo Clinic, Rochester, MN, USA; ^8^Holden Comprehensive Cancer Center, University of Iowa, Iowa City, IA, USA; ^*^These authors contributed equally to this work

**Keywords:** sarcoma, YAP, TAZ, p53-MDM2, RABL6A

## Abstract

Additional prognostic and therapeutic biomarkers effective across different histological types of sarcoma are needed. Herein we evaluate expression of TAZ and YAP, the p53-MDM2 axis, and RABL6A, a novel oncoprotein with potential ties to both pathways, in sarcomas of different histological types. Immunohistochemical staining of a tissue microarray including 163 sarcomas and correlation with clinical data showed that elevated YAP and TAZ independently predict worse overall and progression-free survival, respectively. In the absence of p53 expression, combined TAZ and YAP expression adversely affect overall, progression free, and metastasis free survival more than TAZ or YAP activation alone. RABL6A independently predicted shorter time to metastasis and was positively correlated with p53, MDM2 and YAP expression, supporting a possible functional relationship between the biomarkers. Network analysis further showed that TAZ is positively correlated with MDM2 expression. The data implicate all five proteins as clinically relevant downstream players in the Hippo pathway. Finally, a novel inhibitor of MDM2 (MA242), effectively suppressed the survival of sarcoma cell lines from different histological types regardless of p53 status. These findings suggest both independent and cooperative roles for all five biomarkers across different histological types of sarcoma in predicting patient outcomes and potentially guiding future therapeutic approaches.

## INTRODUCTION

Sarcomas are difficult to treat malignant mesenchymal neoplasms arising in bone or soft tissue. Surgical resection is the mainstay of therapy for localized sarcomas; however, this is not always possible if the sarcoma arises adjacent to crucial anatomical structures. Few effective medical therapies are available for metastatic sarcoma and the average five year survival for metastatic sarcoma remains at 15% [1]. Over 50 histological types of sarcoma have been identified and many of these demonstrate widely divergent clinical behavior [2]. Although the French (FNCLCC) and NCI grading schemes have been adopted for many sarcomas [2, 3], there is a substantial subset of sarcomas for which the grading scheme does not adequately predict clinical behavior. For these reasons, additional therapeutic targets and prognostic markers that would be effective across different histological types of sarcoma are needed.

The Hippo pathway represents such a therapeutic target. Hippo signaling controls the growth, development and regeneration of mammalian tissues and has been implicated in a number of different cancers [4]. The pathway is composed of a series of serine/threonine kinases, MST1/2 and LATS1/2, and its transcriptional effectors, TAZ (transcriptional coactivator with PDZ binding motif) and YAP (Yes associated protein). In normal cells, during conditions of confluence or detachment, the Hippo pathway is activated resulting in phosphorylation of TAZ and YAP by LATS1/2. When TAZ/YAP are phosphorylated, the equilibrium of these transcriptional coactivators is shifted out of the nucleus into the cytoplasm where they subsequently undergo ubiquitin-mediated degradation and are inactivated. TAZ and YAP are oncogenic transcriptional coactivators that activate a transcriptional program promoting cell proliferation and anchorage-independent growth [4]. Hippo pathway dysregulation is an important impetus for uncontrolled cell growth or neoplasia. However, compared to other signaling pathways involved in cancer, few somatic or germline mutations have been discovered in Hippo pathway genes [4].

Studies have found that TAZ (encoded by *WWTR1*) and YAP are constitutively activated and located in the nucleus in various carcinomas including breast [5], colon [6], liver [7], lung [8], pancreas [9], and thyroid [4, 10]. More recently, TAZ/YAP were shown to be activated in multiple histological types of sarcoma [11–13]. In epithelioid hemangioendothelioma (EHE), a vascular sarcoma, a *WWTR1*-*CAMTA1* gene fusion encodes a constitutively activated form of TAZ (TAZ-CAMTA1 fusion protein) that activates a TAZ-like transcriptional program [14–16]. While this fusion protein demonstrates that TAZ/YAP, in some contexts, can initiate sarcomagenesis, what is not known are the genetic hits that cooperate with TAZ activation to promote sarcomagenesis.

One putative cooperative genetic event is loss of p53 activity. The *TP53* gene encodes for the p53 protein, a key tumor suppressor known as the guardian of the genome [17]. Normal cell division is controlled at different checkpoints to avoid inappropriate or aberrant cell growth. If a cell has sustained DNA damage or stress, p53 expression is upregulated and the cell undergoes senescence or apoptosis [18, 19]. Alterations of the p53 pathway are among the most frequent aberrations observed in human cancers, including sarcomas [20, 21]. p53 appears to be an essential player in sarcomagenesis. Individuals with Li-Fraumeni syndrome (LFS) have inherited mutations in *TP53* and are prone to the development of multiple tumor types at an early age. Among the most common type of tumors noted in individuals with LFS are sarcomas, specifically soft tissue sarcomas and osteosarcomas [22]. Alterations in the p53 pathway also appear to confer a metastatic advantage with regard to sarcomas [23] and thus a poor overall survival and prognosis [24].

Mouse double minute 2 homolog (MDM2) is an E3 ubiquitin ligase and powerful negative regulator of p53. MDM2 directly binds to an N terminal phosphodegron in p53, thereby inactivating the protein by promoting its degradation [25]. As such, the amplification of the *MDM2* gene region in several sarcomas including well-differentiated liposarcoma/dedifferentiated liposarcoma, parosteal and low-grade central osteosarcoma, and intimal sarcomas represents an effective mechanism of p53 inactivation [26–32]. Thus, MDM2 is part of a p53-MDM2 axis known to play a key role in several sarcomas.

Other ways of activating MDM2 besides genetic amplification also exist [33, 34], including upregulation at the protein level by a novel RAB-like GTPase called RABL6A. RABL6A is a newly recognized oncoprotein that has been implicated in various human cancers, including pancreatic neuroendocrine tumors [35, 36], breast cancer [37, 38], colon cancer [37], non-small cell lung cancer [39, 40], pancreatic ductal adenocarcinomas [41] and osteosarcoma [42]. In pancreatic neuroendocrine tumors, RABL6A activates Akt signaling through PP2A inactivation and drives G1 to S phase progression via inactivation of the retinoblastoma (RB1) tumor suppressor [35, 36]. Negative regulation of RB1 signaling by RABL6A has also been observed in osteosarcoma [42]. Most recently, RABL6A was shown to be an essential driver of a highly aggressive sarcoma, malignant peripheral nerve sheath tumors (MPNSTs) [43]. Because RABL6A functionally interacts with the Alternative Reading Frame (ARF)-Mdm2-p3 tumor suppressor pathway [34, 44], we hypothesized that it may be a key prognostic marker in other sarcomas and potentially interact with the other biomarkers being evaluated.

## RESULTS

### Biomarker patterns of expression in various sarcomas

**Figure 1:**
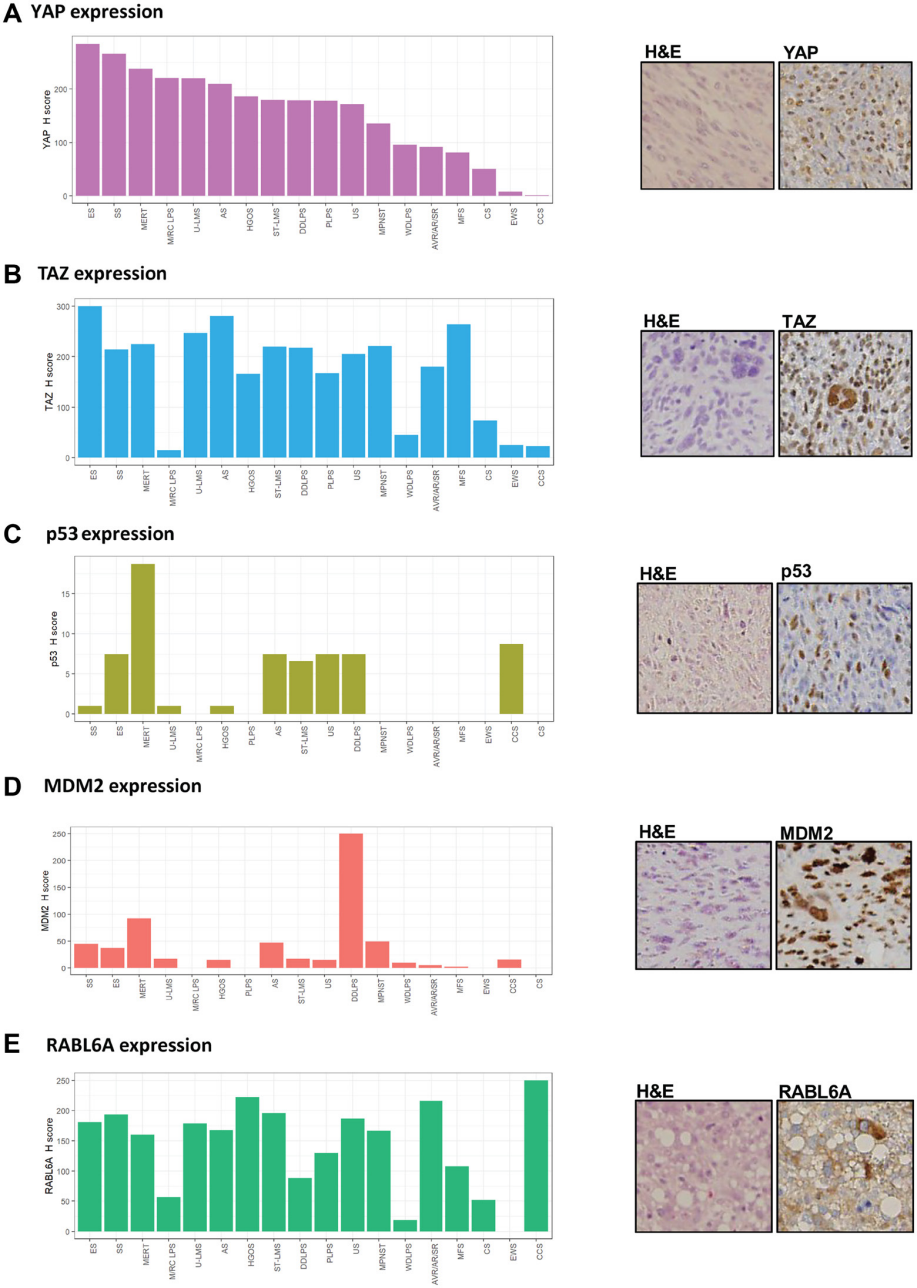
YAP, TAZ, p53, MDM2 and RABL6A are widely expressed in various sarcomas. (**A**) Median H-scores for YAP expression across histological types in descending order of expression. Histological section of an undifferentiated spindle cell sarcoma with YAP nuclear expression by immunohistochemistry (IHC). (**B**) Median H-scores for TAZ expression arranged in similar order to (A); histological section of an undifferentiated pleomorphic sarcoma with TAZ nuclear expression by IHC. (**C**) Median H-scores for p53 expression in sarcomas. Histological section of an undifferentiated spindle cell sarcoma with nuclear p53 expression by IHC. (**D**) Median H-scores for MDM2 expression in sarcomas. Histological section of dedifferentiated liposarcoma with MDM2 nuclear expression by IHC. (**E**) Median H-scores for RABL6A expression in sarcomas. Histological section of pleomorphic liposarcoma with RABL6A cytoplasmic localization. ES = epithelioid sarcoma; SS = synovial sarcoma; MERT = malignant extrarenal rhabdoid tumor; M/RC LPS = myxoid/round cell liposarcoma; U-LMS = uterine leiomyosarcoma; AS = angiosarcoma; HGOS = high grade osteosarcoma; ST-LMS = soft tissue leiomyosarcoma; DDLPS = dedifferentiated liposarcoma; PLPS = pleomorphic liposarcoma; US = undifferentiated sarcoma (undifferentiated pleomorphic sarcoma/undifferentiated spindle cell sarcoma); MPNST = malignant peripheral nerve sheath tumor; WDLPS = well-differentiated liposarcoma; AVR/AR/SR=alveolar rhabdomyosarcoma, adult-type rhabdomyosarcoma, sclerosing/spindle cell rhabdomyosarcoma; MFS = myxofibrosarcoma; CS = chondrosarcoma; EWS = Ewing sarcoma; CCS = clear cell sarcoma of soft parts.

The protein expression of each candidate biomarker of sarcoma (TAZ, YAP, p53, MDM2 and RABL6A) was examined by immunohistochemical (IHC) staining in a clinically annotated tissue microarray (TMA) containing 163 sarcomas representing 18 different histologic subtypes [11]. To facilitate statistical analysis of the IHC results, H-scores (intensity x % positive cells) were calculated for each protein (Supplementary Table 1 and Figure 1A–1E). The median H-score for YAP was 164 (IQR 68-255). Sarcomas with the highest YAP expression included epithelioid sarcoma, synovial sarcoma, and malignant extrarenal rhabdoid tumor (Figure 1A). The median H-score for TAZ was 200 (IQR 80-270) with the greatest expression levels observed in epithelioid sarcoma, angiosarcoma, and myxofibrosarcoma (Figure 1B). Importantly, YAP and TAZ expression did not entirely mirror one another, suggesting differences in their regulation.

As a powerful tumor suppressor, wild-type p53 expression is either low to undetectable in normal cells and tissues. Strong detection of p53 in tumors typically correlates with *TP53* genetic mutations that impair MDM2-mediated degradation of the protein [45]. The presence of `wild-type staining’ in some tissues has been described as an admixture of negative cells, weakly, and strongly positive cells, with the latter likely reflecting its natural stress-induced stabilization. On the other hand, diffuse and strong nuclear staining of p53 is highly correlated with its mutation [46–49]. Because tiered scoring systems of p53 used to calculate the likelihood of p53 mutation introduce an element of arbitrariness to the evaluation, we utilized an unbiased approach where all p53 staining was considered in the statistical evaluation. The median expression value of p53 was 0 (IQR 0-8), with expression of the protein detected in 45.8% of samples. Sarcomas demonstrating p53 expression (Figure 1C) included malignant extrarenal rhabdoid tumor, epithelioid sarcoma, undifferentiated sarcoma (undifferentiated pleomorphic/spindle cell sarcoma), angiosarcoma, high grade osteosarcoma, and dedifferentiated liposarcoma. Of note, dedifferentiated liposarcoma (DDLPS) displayed elevated levels of p53 compared to well-differentiated liposarcoma (WDLPS), which is consistent with tumor progression and served as an internal control.

By comparison, MDM2 was highly expressed in malignant extrarenal rhabdoid tumor, dedifferentiated liposarcoma-well differentiated liposarcoma (known to harbor amplification of *MDM2* [50]), and malignant peripheral nerve sheath tumor (Figure 1D). Higher levels of MDM2 expression were also seen in DDLPS relative to WDLPS, as has been previously described, thus providing further validation for the data set [50]. The median expression value of MDM2 was 15 (IQR 0-62).

RABL6A expression was relatively evenly distributed across histological types (Figure 1E) with a median H score of 150 (IQR 65-218). This included sarcomas where RABL6A has been shown to play a key role in their pathogenesis, such as malignant peripheral nerve sheath tumor [43], as well as other poorly differentiated sarcomas lacking reliable biomarkers such as undifferentiated sarcoma (undifferentiated pleomorphic/spindle cell sarcoma) and pleomorphic liposarcoma. The highest levels of RABL6A were observed in clear cell sarcoma (CCS), one of the rarer sarcomas associated with poor prognosis due to metastatic spread and recurrence.

### Biomarker associations with sarcoma pathological stage and grade

Potential correlations between expression of the various biomarkers with clinical behavior and prognosis were examined, combining histological types, with an initial focus on pathological stage and grade. Pathological staging in sarcomas utilizes the American Joint Committee on Cancer (AJCC) TNM system [51]. Unadjusted univariate linear regression analysis suggested a negative association between tumor size (*T* stage) and both RABL6A and TAZ expression. When adjusting for age, gender, and tumor type, these associations are no longer significant.

**Figure 2:**
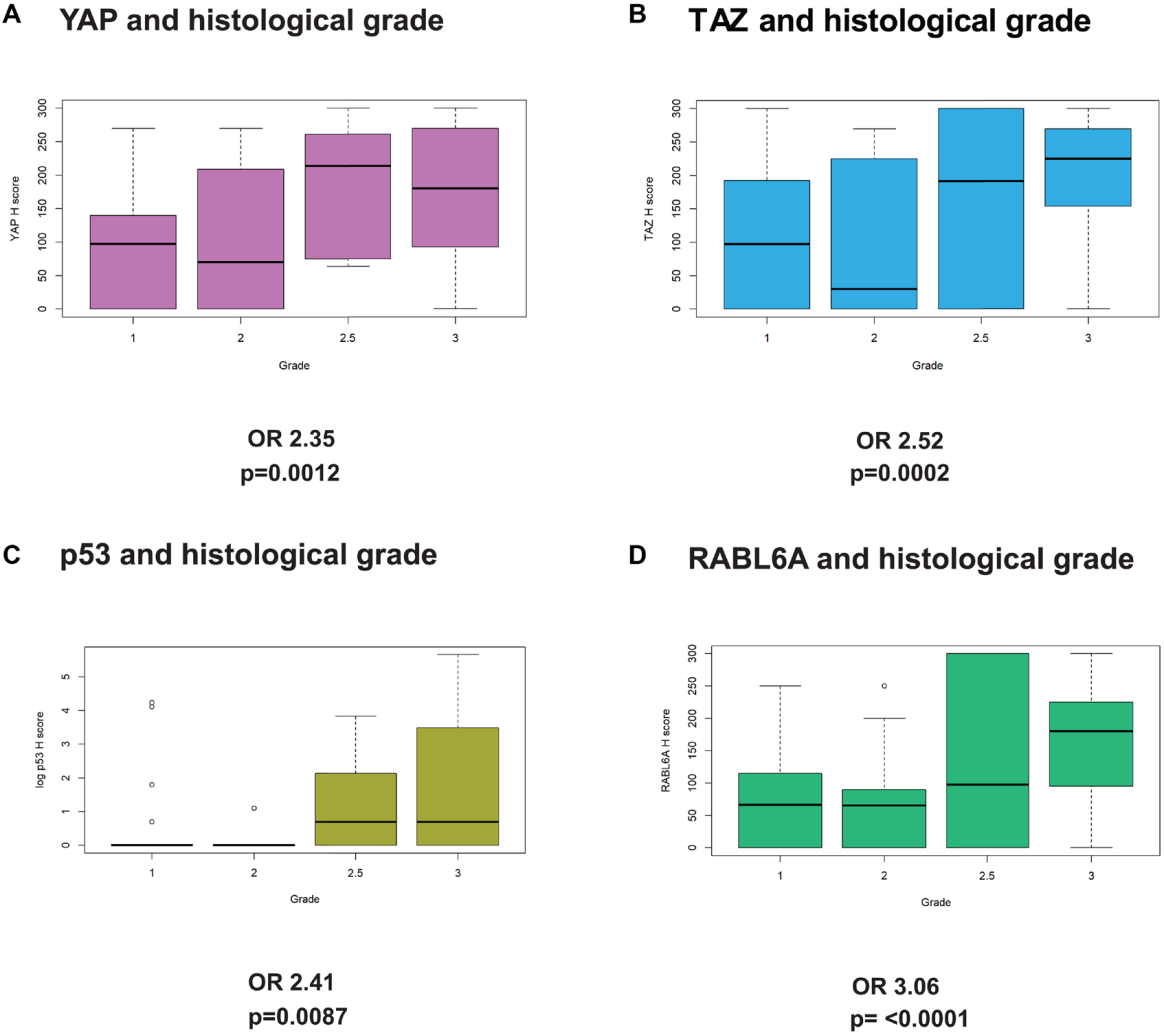
YAP, TAZ, p53, and RABL6A expression are associated with higher histological grade. Unadjusted logistic regression showed (**A**) YAP, (**B**) TAZ, (**C**) p53, and (**D**) RABL6A expression are associated with higher histological sarcoma grade. Grade 1 = low grade, Grade 2 = intermediate grade, Grade 2.5 = intermediate to high grade, and Grade 3 = high grade. Box plots demonstrate the sample median, interquartile range, and outliers if present.

YAP and TAZ levels have been shown to correlate with higher histological grade or prognosis in a number of different carcinomas [6, 52], but their role in predicting grade in sarcomas was previously limited to a few histological types [11]. Similar to other cancers, both YAP and TAZ protein expression were significantly associated with histological grade based on unadjusted univariate logistic regression (Figure 2A and 2B). TAZ demonstrated a slightly higher odds ratio (OR) of 2.52 (*p* = 0.0002) than YAP (OR = 2.35; *p* = 0.0012) and, among the five biomarkers, uniquely demonstrated a significant OR after adjusted univariate analysis (OR 4.75; *p* = 0.0084).

Mouse studies have established a biologically significant role for p53 inactivation in sarcoma tumor progression [53–55], but its role in predicting histological grade in sarcomas has not been well-defined. We found p53 H-scores to be positively associated with grade (OR = 2.41; *p* = 0.0087) as a function of histological grade with an OR of 2.41 (Figure 2C). Similar to p53, RABL6A has also been shown to promote the tumor progression of malignant peripheral nerve sheath tumor [43] which led us to hypothesize that its expression may be associated with histological grade. Indeed, unadjusted univariate analysis showed that of the 5 biomarkers, RABL6A had the strongest association with tumor grade (Figure 2D, OR = 3.06, *p* < 0.0001).

### YAP and TAZ levels predict worse overall and progression free survival

**Figure 3:**
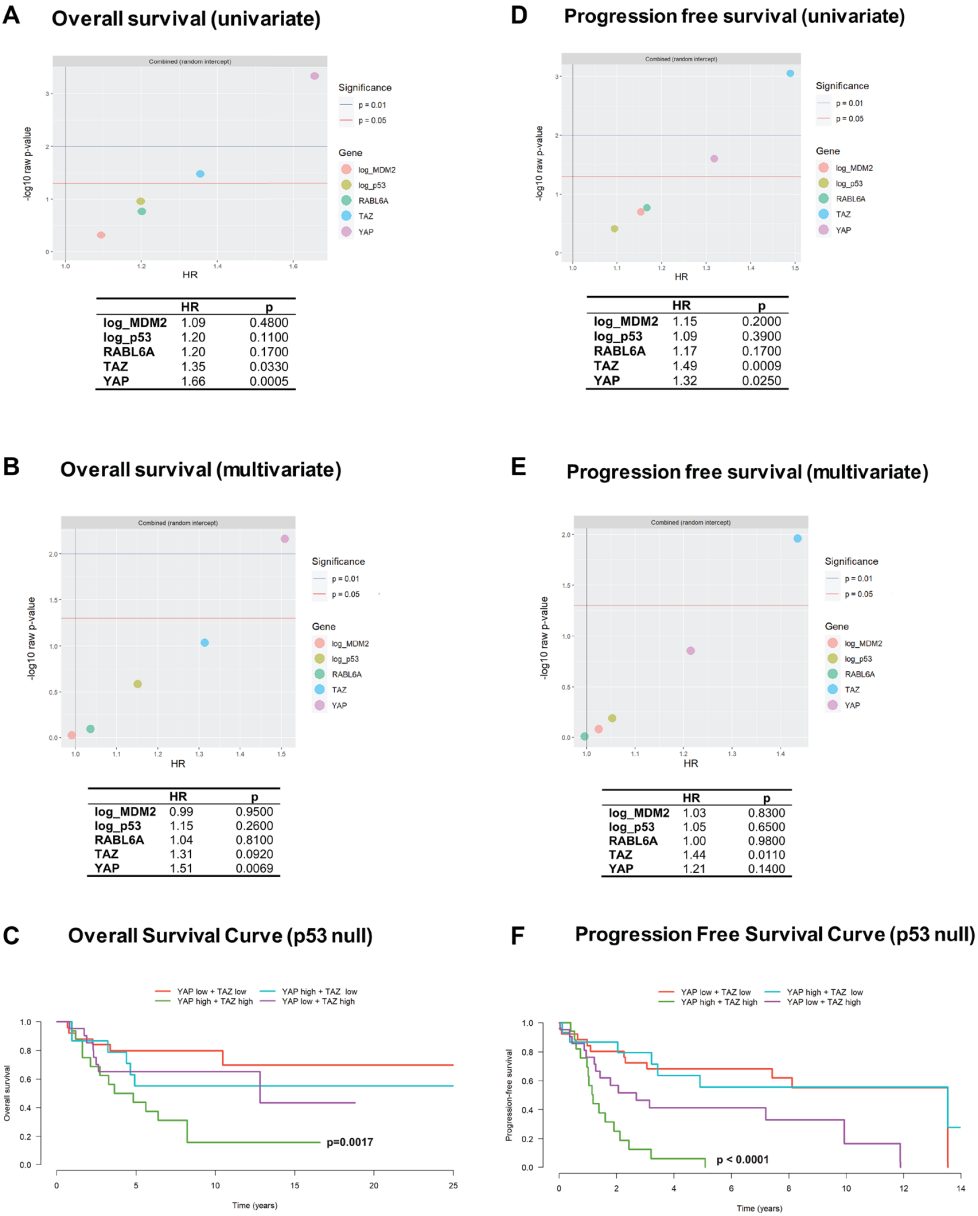
YAP and TAZ are associated with overall and progression free survival across histological types of sarcoma. (**A**) Separate univariate analyses for each biomarker showed YAP and TAZ expression are associated with poorer overall survival. (**B**) A multivariate model including all five biomarkers showed YAP is independently associated with overall survival. (**C**) Kaplan-Meier curves and log-rank rest showed combined high TAZ and YAP expression is associated with poorer overall survival in sarcomas lacking p53 expression. (**D**) Separate univariate analysis for each biomarker demonstrated TAZ and YAP are associated with poorer progression free survival. (**E**) A multivariate model including all five biomarkers showed TAZ is independently associated with progression free survival. (**F**) Kaplan-Meier curves and log-rank test showed combined high TAZ and YAP expression is associated with poorer progression free survival in sarcomas lacking p53 expression.

We have previously shown that higher mRNA expression of *WWTR1* and *YAP1* portend a poorer prognosis with shorter overall survival in two histological types, undifferentiated pleomorphic sarcoma and dedifferentiated liposarcoma [11]. To determine if TAZ, YAP, or the other biomarkers predicted poorer overall survival in other histological types of sarcoma, we performed Cox regression and log-rank tests using H-scores from the various samples and the linked survival data (Supplementary Figure 1A–1C and Supplementary Tables 1–4). Adjusted univariate analysis showed that YAP (HR = 1.66; *p* = 0.0005) and TAZ (HR = 1.35; *p* = 0.033) predicted a poorer overall survival (Figure 3A). Moreover, multivariate analysis showed that YAP alone independently predicted poorer overall survival (HR = 1.51; *p* = 0.0069) (Figure 3B). Because p53 plays a key role in sarcoma pathogenesis [22–24] we dichotomized the sarcomas into p53 positive (Supplementary Figure 1D) and p53 null groups (Figure 3C), and further evaluated the role of TAZ and YAP in determining overall survival. In the p53 null group, combined TAZ and YAP expression (nuclear localization indicating activated forms) determined markedly poorer overall survival that was statistically significant (*p* = 0.0017). The cooperative effect of TAZ and YAP expression predicting worse overall survival can be explained in part by the univariate analysis (Figure 3A), which shows that TAZ has a statistically significant albeit lower hazard ratio than YAP.

We then determined if expression of these five biomarkers correlated with progression free survival (defined as time from diagnosis to first local recurrence or metastasis). In contrast to overall survival, adjusted univariate analysis showed that TAZ (HR = 1.49; *p* = 0.0009) rather than YAP (HR = 1.32; *p* = 0.0250) had the higher hazard ratio (Figure 3D), suggesting a more prominent role in predicting progression free survival. This was further validated by the adjusted multivariate analysis (Figure 3E) which showed that TAZ alone (HR = 1.44; *p* = 0.0110) directly predicted progression free survival. Splitting the sarcomas into p53 positive (Supplementary Figure 1E) and null groups (Figure 3F) showed a similar finding to overall survival (Figure 3C) with combined TAZ and YAP portending a much poorer prognosis across all sarcoma types. This finding is also consistent with the observation by univariate analysis showing that TAZ and YAP separately correlated with reduced progression free survival in a statistically significant manner (Figure 3D).

### RABL6A expression predicts metastasis free survival

**Figure 4:**
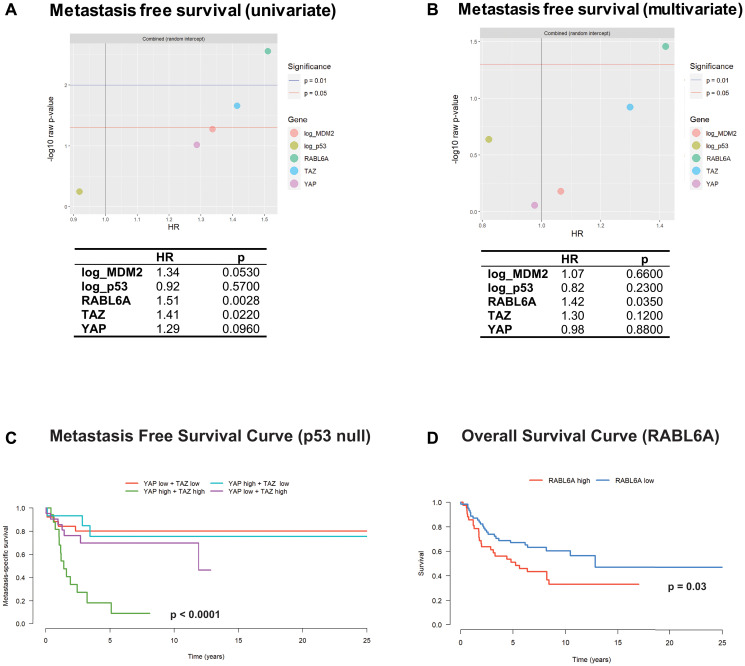
RABL6A and the Hippo pathway axis predict metastasis free survival. (**A**) Separate analyses for each biomarker show that RABL6A and TAZ are associated with a shorter time to metastasis. MDM2 trends towards statistical significance (*p* = 0.0530). (**B**) A model including all five biomarkers showed that RABL6A is independently associated with poorer metastasis free survival. (**C**) Kaplan-Meier curves and log-rank test showed combined high TAZ and YAP expression is associated with a worse metastasis free survival in sarcomas lacking p53 expression. (**D**) Kaplan-Meier curves and log-rank test show RABL6A is associated with poorer overall survival when evaluating the upper and low tertiles.

Metastasis, typically pulmonary metastasis, is the main cause of mortality in sarcoma patients [56]. Biomarkers correlating with metastasis in sarcomas could potentially play an important role in guiding clinical management. Because TAZ was found to be a prognostic determinant of progression free survival, we sought to determine if TAZ and potentially other biomarkers would predict metastasis-free survival. Indeed, adjusted univariate analysis of metastasis free survival (Figure 4A) revealed that TAZ expression/activation predicted a statistically significant (*p* = 0.0220) worse metastasis free survival (HR = 1.41). RABL6A had a slightly higher hazard ratio (HR = 1.51; *p* = 0.0028) than TAZ by univariate analysis and was the only protein that independently predicted poorer metastasis free survival by multivariate analysis (HR = 1.42; *p* = 0.0350) (Figure 4B). MDM2 (Figure 4A), but not p53 expression, trended towards predicting worse metastasis free survival (HR = 1.34, *p* = 0.0530). As in the overall and progression free survival analyses, we observed evidence of interactions between p53 and other proteins. Specifically, high TAZ and YAP levels predicted poorer metastasis free survival in the p53 null setting (Figure 4C) but not in tumors expressing p53 (Supplementary Figure 1F).

The significant, independent effect of RABL6A on metastasis free survival (Figure 4B) prompted us to further explore its role in overall survival. A log-rank test comparing the highest and lowest tertiles of RABL6A expression (Figure 4D) demonstrated that high RABL6A expression portended a poorer prognosis in sarcomas compared to those with low RABL6A expression (*p* = 0.03). Additional analysis showed that increased RABL6A expression co-segregated with more aggressive histological types of sarcoma (Supplementary Figure 2A), indicating that the association between RABL6A illustrated in Figure 4D is driven by RABL6A’s high expression in aggressive sarcomas, rather than RABL6A expression within a given tumor type. This explains why a statistically significant HR was not identified in the initial analysis (Figure 3A and 3B), which quantifies association with survival within tumor type.

### An integrated model of Hippo pathway, p53/MDM2, and RABL6A signaling

**Figure 5:**
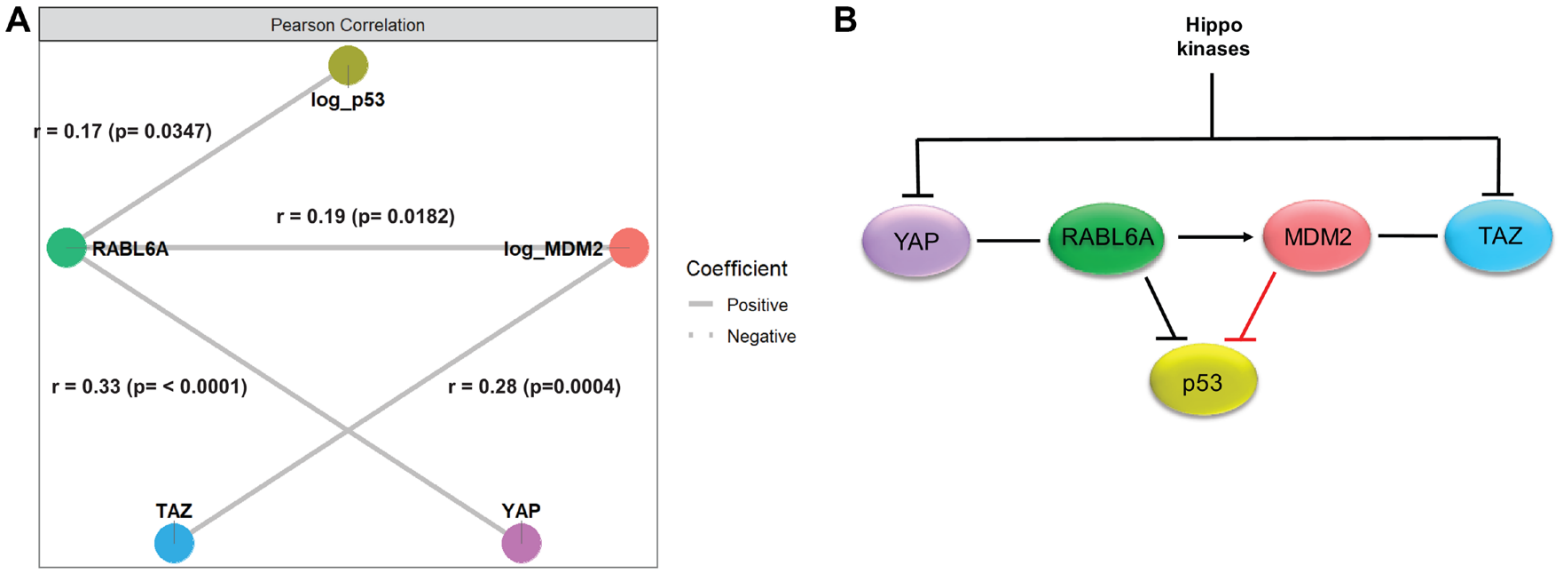
Network analysis supported a model integrating the Hippo pathway, p53/MDM2 axis and RABL6A signaling in sarcomas. (**A**) Correlation-based network analysis showed statistically significant, positive correlations between RABL6A and p53, MDM2, and YAP. In addition, a statistically significant positive correlation was identified between TAZ and MDM2. (**B**) Model diagram depicting the relationship between the five biomarkers integrating the network analysis and previously identified functions of the proteins, with RABL6A serving as a potential link between YAP and TAZ and integrator of the p53-MDM2 axis.

To explore the relationships between the Hippo pathway, the p53/MDM2 axis, and the RABL6A axis further, we performed correlation-based network analysis (Figure 5A). RABL6A and p53 were positively correlated (*r* = 0.17; *p* = 0.0347) as were RABL6A and MDM2 (*r* = 0.19; *p* = 0.0182), mirroring interactions that have been previously described [34, 35]. Previously undescribed positive correlations between YAP and RABL6A (*r* = 0.33; *p* < 0.0001) and TAZ and MDM2 (*r* = 0.28; *p* = 0.0004) were also identified, linking the Hippo pathway to RABL6A and the p53/MDM2 axis. To identify direct protein-protein associations, Gaussian graphical models were also used (Supplementary Figure 2B). These analyses continued to show positive associations between RABL6A and p53 (coef = 0.27; *p* = 0.0120) and RABL6A with YAP (coef = 0.33; *p* = 0.0003), indicating an association of these proteins independent of the expression levels of the other biomarkers. These analyses also indicated a direct positive association between TAZ and MDM2 expression levels (coef = 0.23; *p* = 0.0441).

Integrating the above network analysis with known functions of the proteins, the following model diagram was constructed (Figure 5B). Both TAZ and YAP are transcriptional coactivators and nuclear effectors of the Hippo pathway that are negatively regulated by the upstream Hippo kinases (MST1/2 and LATS1/2) [57–65]. As indicated in the network analysis, RABL6A is positively correlated with YAP, while MDM2 is positively correlated with TAZ. RABL6A is known to drive MDM2 activity and, as mentioned above, its expression is positively correlated with MDM2. In this way, the correlation analysis suggests RABL6A represents a previously unappreciated link between YAP and TAZ. RABL6A is positively correlated with dysregulated p53 (likely including mutated p53 in clinical samples with the highest H-scores) but is known to inhibit the function of the wild-type tumor suppressor [34], as such the relationship between the two proteins is indicated by an inhibitory sign. Although MDM2 is known to negatively regulate p53, a negative correlation was not observed by immunohistochemical (IHC) approaches. This likely reflects the fact that IHC predominantly detects mutant p53, which is known to demonstrate defects in transcriptional upregulation of MDM2 [66].

### Pharmacological inhibition of MDM2 can be used to target sarcoma cell lines overexpressing MDM2 independent of p53 status

We and others have previously shown that verteporfin can inhibit the YAP-TEAD interaction in sarcomas and suppress their growth [11, 13, 67–70]. In this study, we attempted to determine whether other biomarkers in the RAB6A/YAP/p53-MDM2 axis can also be therapeutically targeted.

**Figure 6:**
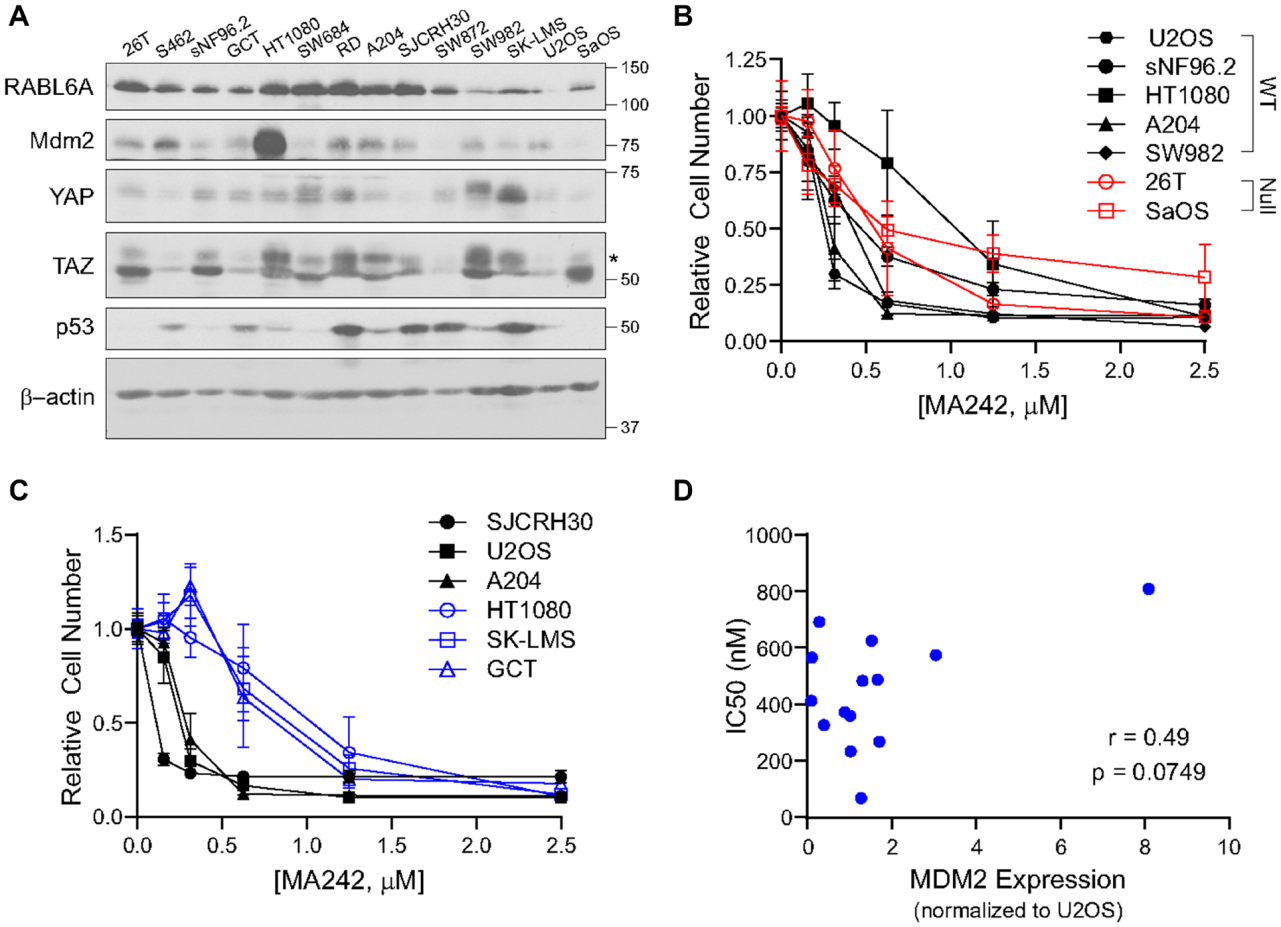
MDM2 can be targeted sarcoma cell lines independent of p53 status. (**A**) Expression of RABL6A, MDM2, YAP, TAZ, and p53 in sarcoma cell lines. (**B**) Cells were treated for 3 days with MA242 and analyzed with AlamarBlue (MTT-style proliferation assay); MA242 similarly inhibits proliferation in p53 wild-type and p53 null sarcoma cell lines. (**C**) Sarcoma cell lines most sensitive (drug response curves—black) and least sensitive (drug response curves—blue) to MA242 treatment. (**D**) Positive correlation of IC50 with MDM2 expression trends towards statistical significance.

Using a panel of 13 mesenchymal neoplasm/sarcoma cell lines [12], we found a broad range of expression for all five proteins of interest (Figure 6A), mirroring their wide distribution of expression in sarcoma clinical samples. No inhibitors of RABL6A currently exist, but various types of MDM2 inhibitors have been developed that either inhibit the MDM2-p53 interaction, its E3 ligase activity, or dampen its expression [71–73]. However, no MDM2 inhibitors are currently in routine clinical use. Herein, we use MA242, a small molecule that ablates MDM2 expression by simultaneously inducing MDM2 auto-ubiquitination and degradation as well as inhibiting NFAT1-mediated *MDM2* transcription independent of p53 status [74, 75]. Its efficacy in sarcomas has not been previously demonstrated, so we evaluated its efficacy in the above cell lines using an MTT-style proliferation assay (Figure 6B, 6C and Supplementary Figure 2C). Consistent with its p53-independent mechanism, MA242 inhibited sarcoma cell lines regardless of whether p53 was wild type or null (Figure 6B).

MA242 was effective at low to high nM doses across the sarcoma lines tested. IC50 values ranged from 67 nM with the SJCRH30 cell line up to 813 nM for the HT1080 cell line (Figure 6C; summarized in Table 1). The observation that HT1080 fibrosarcoma cells demonstrated both the highest IC50 and MDM2 expression level prompted us to determine whether a correlation between the IC50 of MA242 and expression of MDM2 existed. Using densitometry, we showed a modest positive correlation (*r* = 0.49) between MA242 IC50 and MDM2 expression (normalized to expression of MDM2 in U2OS) that trended toward statistical significance (*p* = 0.0749) (Figure 6D). This finding is consistent with the mechanism of MA242, which depletes MDM2 levels [74]. This also suggests that expression of MDM2 could potentially be used to predict response to MA242-based therapy.

**Table 1:** Overview of sarcoma cell lines, p53/MDM2 status, and IC50 (MA242)

Cell Line	Sarcoma Subtype	IC50 (nM)	p53 Status	Relative MDM2 Expression
U2OS	Osteosarcoma	234	WT	1.0^*^
SaOS	Osteosarcoma	414	Null	0.1
26T	MPNST	485	Null	1.3
S462	MPNST	577	Mut	3.1
sNF96.2	MPNST	360	WT	1.0
GCT	Giant Cell Tumor	630	Mut	1.5
HT1080	Fibrosarcoma	813	WT	8.1
SW684	Fibrosarcoma	327	Mut	0.4
RD	Embryonal rhabdomyosarcoma	491	Mut	1.7
A204	Malignant extrarenal rhabdoid tumor	269	WT	1.7
SJCRH30	Alveolar rhabdomyosarcoma	67	Mut	1.3
SW872	Liposarcoma	570	Mut	0.1
SW982	Synovial Sarcoma	373	WT	0.9
SK-LMS	Leiomyosarcoma	696	Mut	0.3

## DISCUSSION

### TAZ and YAP demonstrate complementary roles in predicting patient outcomes

Previously, it was shown that *WWTR1* and *YAP1* genetic status can predict overall survival and grade in a few selected types of sarcoma [11]. Our data now establish that TAZ and YAP protein expression positively correlates with histological grade and predicts overall as well as progression free survival across multiple different histological types of sarcomas. Elevated TAZ is a stronger predictor of worse progression-free survival whereas increased YAP better predicts overall survival, implying different functional roles for TAZ and YAP in sarcoma pathogenesis. In that regard, TAZ and YAP are paralogues that share significant identify, particularly within their functional domains [76]. While YAP is conserved down to *Drosophila*, TAZ has more recently emerged in vertebrates [76], suggesting that YAP and TAZ may have similar yet non-overlapping functions. We recently verified distinct, albeit related, activities of TAZ and YAP in sarcoma *in vitro* [11]. Our studies herein confirm that TAZ and YAP drive different phenotypes in the clinical setting and may be used as prognostic biomarkers. Additional studies *in vivo* are warranted to dissect the differential mechanistic contributions of TAZ and YAP to tumorigenesis and metastasis in sarcomas.

### TAZ/YAP are linked to the p53-MDM2 axis

Our data show that p53 expression is also positively correlated with histological grade, in agreement with the observation that genetic alterations involving p53 are frequent in sarcomas [77, 78]. Network analysis showed that YAP is indirectly linked to p53 via RABL6A, while TAZ and MDM2 are positively correlated, indicating that both nuclear effectors of the Hippo pathway interact with the p53-MDM2 axis. TAZ and YAP were prognostically important in p53 null but not p53 high (mutant) sarcomas with regards to overall survival, progression free survival, and metastasis free survival. These data suggest that TAZ and YAP may cooperate with loss of p53 expression in some contexts to drive sarcomagenesis. This is consistent with several studies that have linked YAP and TAZ to p53 in various capacities, either by direct interaction or at a functional level [79]. Additional studies are needed to evaluate how inactivation of p53 cooperates with TAZ and YAP activation in sarcoma tumor progression. Importantly, immunohistochemical panels containing p53, TAZ, and YAP may be valuable in identifying sarcoma patients anticipated to have a particularly aggressive clinical course.

### The RABL6A oncoprotein is a potential link between the TAZ/YAP and p53-MDM2 axis

Our data show that RABL6A expression predicted histological grade, likely connected to its prognostic role predicting metastasis-free survival, and confirmed its role as an oncoprotein across different histological types of sarcoma. One of the questions the above findings raise is whether TAZ/YAP activation, inactivation of p53, and activation of MDM2 are entirely stochastic events or mechanistically linked. Because RABL6A functionally interacts with p53 and MDM2 [34], we hypothesized it may connect the different signaling axes. Multivariate network analysis showed a positive correlation between RABL6A with both YAP and p53 expression. Whether or not RABL6A contributes to dysregulation of those signaling pathways in a GTPase-dependent manner is an important question for future functional studies.

RABL6A expression was recently shown to be greatly upregulated in MPNSTs relative to benign, patient-matched plexiform neurofibromas [43]. Moreover, atypical neurofibromatous neoplasm of uncertain biological potential (ANNUBP), an intermediate step in tumor progression between plexiform neurofibromas and MPNST, expressed intermediate levels of RABL6A, directly correlating its expression with MPNST progression. The findings suggest RABL6A is activated at the ANNUBP step, where its ability to inactivate the cell cycle inhibitor, p27, is predicted to accelerate cell cycle progression as it does in MPNST [43]. Here, the discovery that RABL6A expression correlates with dysregulated p53 and YAP across diverse sarcoma types suggests a key role for RABL6A in promoting the pathogenesis of other sarcomas. Additional studies determining the mechanisms by which RABL6A regulates the activity of YAP and p53 in sarcomas are warranted.

### Clinical applications of the RABL6A, TAZ/YAP, p53-MDM2 network

The interrelated RABL6A/YAP/p53 and TAZ/MDM2 network provides multiple opportunities for therapeutic intervention. RABL6A has been targeted indirectly by inhibiting cyclin dependent kinases 4/6 and 2 in MPNST [43]. We and others have shown that TAZ/YAP can be targeted in sarcomas with verteporfin, which targets the TAZ/YAP-TEAD interaction [11, 13, 67–70]. p53 has shown the potential for therapeutic targeting [80]. MDM2 inhibitors have been developed that target MDM2 in the context of wild-type p53. In this study, we demonstrate that a novel MDM2 inhibitor, MA242, with a p53-independent mechanism [74, 75] can also be used to target this network. MA242 was effective in the nM range in a number of different sarcoma cell lines, suggesting that MDM2 is a relevant, common therapeutic target for suppressing different histological types of sarcoma. We expect that combination therapies targeting this network will be most effective in the treatment of sarcomas. Furthermore, there is a need to further validate these RABL6A/YAP/p53 and TAZ/MDM2 expression signatures in larger numbers of different histological types of sarcoma to determine if it differentially predicts prognosis or response to therapy within individual subsets of these sarcomas. We anticipate these above efforts will lead to a more effective, tailored approach for these cancers for which few effective medical therapies are currently available.

## MATERIALS AND METHODS

### Tissue microarray construction

A total of 159 untreated sarcomas were retrieved from the University of Iowa Department of Pathology and clinical data obtained with previous approval from the Institutional Review Board. The tissue microarray was constructed by arraying 1.0 mm cores taken from formalin fixed paraffin embedded tissue and assembled using a MTA-1 tissue arrayer from Beecher Instruments (Sun Prairie, WI) as previously described [11]. Sarcomas were classified according to World Health Organization criteria [2].

### Clinical data

Institutional Review Board approval was obtained prior to collection of clinical data. Histological grade for soft tissue sarcomas reported in the clinical data analyzed in this study utilized the National Cancer Institute (NCI) grading scheme which depends on the number of mitoses per high-power field, the presence of necrosis, cellular and nuclear morphology, and the degree of cellularity [3].

### Antibodies for immunohistochemistry

Anti-YAP (rabbit polyclonal, catalog #sc-15407) utilized for immunohistochemistry (1:100) was obtained from Santa Cruz Biotechnology (Santa Cruz, CA, USA). Anti-TAZ (mouse monoclonal 1H9; catalog # LSC173295) utilized for immunohistochemistry (1:50) was obtained from LifeSpan Biosciences (Seattle, WA, USA). Anti-p53 (mouse monoclonal DO7, catalog #M7001) utilized for immunohistochemistry (1:100) was obtained from Dako (Agilent) (Santa Clara, CA, USA). Anti-MDM2 utilized for immunohistochemistry (mouse monoclonal OP46; catalog# OP46-100UG) was obtained from Millipore (CalBiochem). utilized for immunohistochemistry Anti-RABL6A utilized for immunohistochemistry [44] was obtained from the Quelle lab (D.E.Q, University of Iowa). See Supplementary Table 5 for additional details regarding antigen retrieval protocols and secondary antibody reagents.

### Antibodies for western blot

Anti-YAP (rabbit monoclonal D8H1X, catalog# 14074) used for western blot (1:1000) was obtained from Cell Signaling (Danvers, MA, USA). Anti-TAZ (rabbit polyclonal, catalog # HPA007415) used for western blot (1:5000) was obtained from Sigma Aldrich (St. Louis, MO). Anti-p53 (DO-1, catalog# sc-126) used for western blot (1:500) was obtained from Santa Cruz Biotechnology. Anti-MDM2 (mouse monoclonal clone 2A10) used for western blot (1:50) was obtained from Oncogene Research Products. Anti-RABL6A (rabbit polyclonal) used for western blot (1.5ug/mL) was obtained from the Quelle lab (D.E.Q, University of Iowa) [44]. β-actin (C-2, catalog# sc-8432) used for western blot (1:500) was obtained from Santa Cruz Biotechnology. Horseradish peroxidase-conjugated secondary antibodies (catalog# NA934 and NA935) were obtained from Sigma.

### Western blot

Harvested cells were counted and lysed in SDS-PAGE loading buffer at 1 × 10^6^c/mL. Identical cell equivalents were electrophoresed through polyacrylamide gels, and proteins were transferred to a polyvinylidene difluoride (PVDF) membrane (Millipore). Membranes were blocked with 5% nonfat milk or 5% BSA in TBST (Tris-buffered saline containing Tween-20) depending on the antibody used to probe (mentioned above). Proteins were detected using HRP-conjugated secondary antibodies and enhanced chemiluminescence (ECL). ImageJ (NIH, Bethesda, MD, USA). was used for densitometry quantification, where a region of interest was defined for each protein and the net protein/net loading control ratios were calculated for each protein and normalized to the U2OS cell lines.

### Proliferation/viability assays with MA242

MA242, a dual MDM2 and NFAT1 inhibitor, was obtained from Dr. Sadanandan Velu and stock solutions stored at –20°C. Sarcoma cell lines were seeded at 1,000 cells per well in 96-well flat-bottom dishes. Varying concentrations of MA242 were added the next day and cells exposed to drug for 3 days. Each condition was performed in triplicate and assayed for relative cell number using AlamarBlue (Thermo Fisher Scientific, DAL1025). Results were quantified using a fluorescence microplate reader by measuring absorbance at 540/570 nm.

### Statistics

#### Data preparation

163 subjects with sarcomas were included for analysis. For each subject and gene, the analyzed H-score ranged from 0 to 300 and was calculated as the mean of two H-scores determined by two pathologists (J.T. and M.R.T.). The clinical covariates gender, age, tumor grade, and tumor size were examined. Tumor size and the H-scores of p53 and MDM2 were highly right-skewed. Because of this, tumor size was log-transformed for all analyses. H-scores of p53 and MDM2 and were log(x+1) transformed, as zeros were present. Tumor grade was excluded from primary analyses due to high levels of missingness (*n* = 60). In a sensitivity analysis which included adjustment for grade, statistically significant associations identified in the primary analysis remained qualitatively consistent, though power to detect statistical significance was reduced to the substantial decrease in sample size. When tumor size was missing (*n* = 13), it was imputed with the median.

### Survival analyses

Cox proportional hazards mixed effect models and the R package coxme were used for all survival analyses. In analyses, sarcoma type was treated as a normally distributed random effect, while clinical covariates and gene H-scores were modeled as fixed effects. These proportional hazards models were used to identify genes associated with various survival outcomes. Overall survival (OS) was defined as the time from diagnosis to patient death, while progression-free survival (PFS) was defined as that from diagnosis to first incidence of disease progression—death, recurrence, or development of metastasis. Metastasis free survival (MFS) or time to metastasis was defined more narrowly as the time from diagnosis to first incidence of metastasis. To explore interactions, dichotomized versions of each gene and their pair-wise interactions were also considered. p53 was dichotomized as present (H-score > 0) or absent (H-score = 0), RABL6A was dichotomized using its upper and lower tertiles, and all other genes were dichotomized as high or low based on their median H-score value.

### Network analyses

Gene-gene networks were constructed to assess the marginal correlation among genes. To adjust for clinical covariates, each gene’s H-score was initially regressed on age, gender, log tumor size, and tumor type. These adjusted expression levels were then used in pairwise Pearson correlation tests. An unadjusted *p*-value of 0.05 was used as a threshold to determine the presence of an edge between genes. Gaussian graphical models were additionally used to evaluate direct associations among genes/proteins. For this analysis, each protein’s H-score was regressed on age, gender, log tumor size, tumor type, and the remaining four proteins. An unadjusted *p*-value of 0.05 was again used as a cutoff to determine the presence of an edge between proteins. All analyses were performed using R version 4.0.3.

## SUPPLEMENTARY MATERIALS




