# Supplementary material for: Prognostic and therapeutic value of the Hippo pathway, RABL6A, and p53-MDM2 axes in sarcomas
Source: Oncotarget. 2021 Apr 13;12(8):740–55. doi: 10.18632/oncotarget.27928 (PMC8057271; doi:10.18632/oncotarget.27928)
Supplement: Supplementary file 1 [file oncotarget-12-740-s001.pdf]

Prognostic and therapeutic value of the Hippo pathway, RABL6A, and p53-MDM2 axes in sarcomas

SUPPLEMENTARY MATERIALS

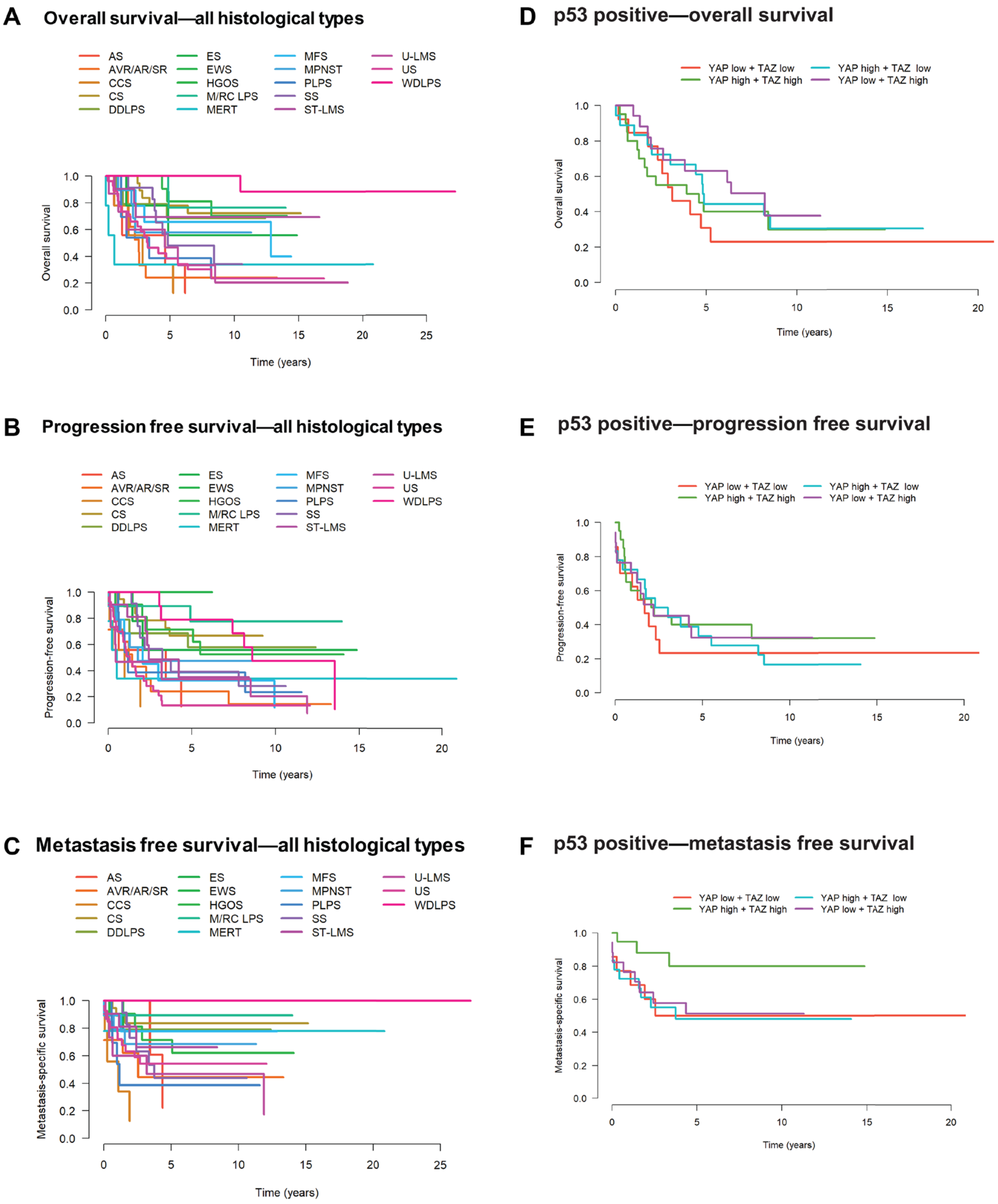

Supplementary Figure 1: Additional Kaplan-Meier analysis validating the data set and evaluating prognostic value of TAZ/YAP expression in p53 high sarcomas. Kaplan-Meier analysis shows overall survival (**A**), progression free survival (**B**), and metastasis free survival (**C**) for various histological types in the data set. Kaplan-Meier curves in the p53 positive setting showing the effect of concurrent high TAZ and YAP expression on overall survival (**D**), progression free survival (**E**), and metastasis free survival (**F**).

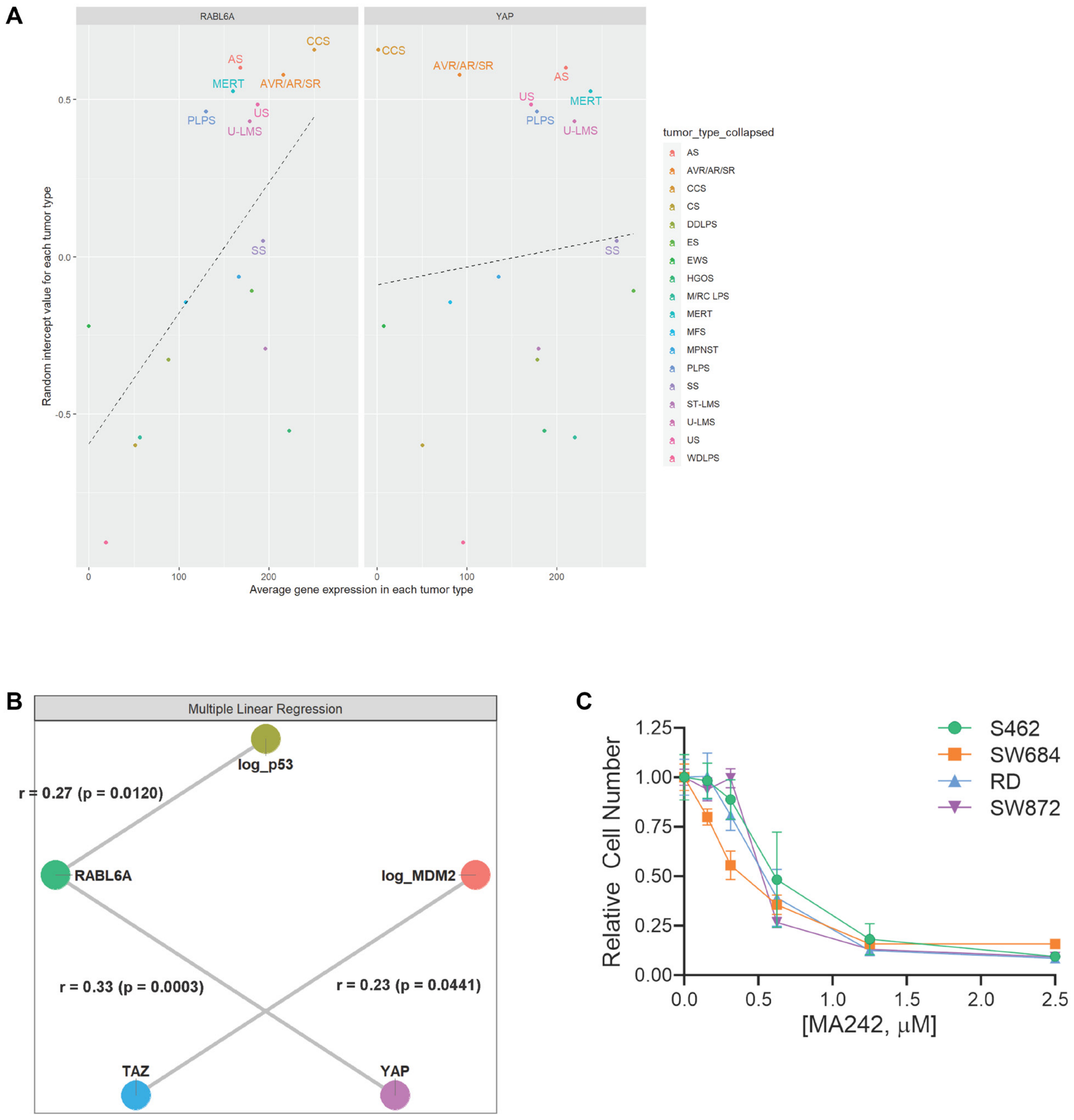

Supplementary Figure 2: Additional analysis of working model and drug response curves for MA242. (**A**) Increased RABL6A expression is associated with histological types demonstrating more aggressive clinical behavior. (**B**) Gaussian graphical model demonstrates direct positive associations of RABL6A with p53 and YAP. A direct positive association is present between TAZ and MDM2. (**C**) Additional drug response curves for the S462, SW684, RD, and SW872 cell lines.

Supplementary Table 1: Summary of clinical data and H-scores for the 5 biomarkers. See Supplementary Table 1

Supplementary Table 2: Median overall survival for different sarcoma histological types

	n	n_events	median	medianYrs_0.95LCL	medianYrs_0.95UCL	survivalProb	survivalProb_0.95LCL	survivalProb_0.95UCL
AS	5	4	4.640657084	0.632443532		0.338465425	0.094516202	1
AVR/AR/SR	10	8	2.568104038	1.73853525		0.239555956	0.081915333	0.700565493
CCS	4	4	2.872005476	0.678986995		0.338465425	0.094516202	1
CS	18	5				0.780119897	0.610942089	0.996145242
DDLPS	9	3		4.772073922		0.684567345	0.444828674	1
ES	4	2		1.590691307		0.558035146	0.246600665	1
EWS	1	0				1	1	1
HGOS	10	3		8.213552361		0.809684097	0.604051535	1
M/RC LPS	9	2				0.765017061	0.527342303	1
MERT	4	3	0.657084189	0.010951403		0.338465425	0.094516202	1
MFS	9	4	12.83230664	3.003422313		0.65662978	0.405413282	1
MPNST	10	4		2.124572211		0.579473747	0.337389859	0.995257604
PLPS	7	5	3.37303217	1.201916496		0.386741023	0.147643214	1
SS	11	7	4.832306639	3.912388775		0.478765668	0.262159544	0.87433996
ST-LMS	10	3		2.32991102		0.692216063	0.454583307	1
U-LMS	7	6	4.594113621	0.97467488		0.467889178	0.218635733	1
US	26	19	3.277207392	2.477754962		0.383358462	0.234407359	0.626958604
WDLPS	9	1				1	1	1

Supplementary Table 3: Median progression free survival for different sarcoma histological types

	n	n_events	median	medianYrs_0.95LCL	medianYrs_0.95UCL	survivalProb	survivalProb_0.95LCL	survivalProb_0.95UCL
AS	5	4	3.433264887	0.569472964		0.124514471	0.012011776	1
AVR/AR/SR	10	9	1.420944559	0		0.239555956	0.081915333	0.700565493
CCS	4	4	0.977412731	0.084873374		0.124514471	0.012011776	1
CS	18	6		3.674195756		0.66777622	0.481739407	0.925656223
DDLPS	9	4		1.256673511		0.579473747	0.337389859	0.995257604
ES	4	2		1.451060917		0.558035146	0.246600665	1
EWS	1	0				1	1	1
HGOS	10	5		2.302532512		0.714543708	0.487711616	1
M/RC LPS	9	2				0.775716428	0.544066945	1
MERT	4	3	0.517453799	0		0.338465425	0.094516202	1
MFS	9	7	2.064339493	0.963723477		0.323366717	0.121420579	0.861188725
MPNST	10	5	1.782340862	0.739219713		0.474432977	0.243259843	0.925293082
PLPS	7	5	1.180013689	0.643394935		0.386741023	0.147643214	1
SS	11	8	3.230663929	1.905544148		0.391980176	0.191066254	0.804163243
ST-LMS	10	6	2.43394935	2.214921287		0.348551859	0.138525802	0.877009165
U-LMS	7	7	0.459958932	0.136892539		0.335257246	0.122984811	0.913913025
US	26	23	1.28678987	0.906228611	2.685831622	0.132511429	0.05011786	0.350359705
WDLPS	9	7	8.616016427	7.436002738		0.789692925	0.568983382	1

Supplementary Table 4: Median metastasis free survival for different sarcoma histological types

	n	n_events	median	medianYrs_0.95LCL	medianYrs_0.95UCL	survivalProb	survivalProb_0.95LCL	survivalProb_0.95UCL
AS	5	2	4.353182752	3.433264887		0.22313016	0.024939401	1
AVR/AR/SR	10	5	2.543463381	0		0.443835364	0.198056636	0.994613632
CCS	4	4	1.092402464	0.084873374		0.124514471	0.012011776	1
CS	18	3				0.837881091	0.685734701	1
DDLPS	9	2				0.789692925	0.568983382	1
ES	4	1		1.451060917		0.778800783	0.477118211	1
EWS	1	0				1	1	1
HGOS	10	4		2.833675565		0.714543708	0.487711616	1
M/RC LPS	9	1				0.894839317	0.719725862	1
MERT	4	1		0		0.778800783	0.477118211	1
MFS	9	2				0.775716428	0.544066945	1
MPNST	10	3		1.571526352		0.684567345	0.444828674	1
PLPS	7	4	1.180013689	0.643394935		0.386741023	0.147643214	1
SS	11	6	3.745379877	2.43394935		0.438045321	0.220859083	0.868806034
ST-LMS	10	3		2.43394935		0.66291327	0.406367724	1
U-LMS	7	5	3.20054757	0.399726215		0.467889178	0.218635733	1
US	26	11		1.626283368		0.541549367	0.372889787	0.786494369
WDLPS	9	0				1	1	1

Supplementary Table 5: Summary of the antigen retrieval methods and secondary antibodies utilized for immunohistochemistry

Protein	Primary antibody	Antigen Retrieval	Secondary Reagents
YAP	Rabbit polyclonal (#sc-15407)	Citrate buffer pH 6.0, 125°C for 5 min; 20 min cool down	Dako Rabbit Envision HRP System (Dako North America, Inc., Carpentaria, CA, USA)
TAZ	Mouse monoclonal 1H9 (# LSC173295)	Citrate buffer pH 6.0, 110°C for 15 min; 20 min cool down	Vector biotinylated anti-mouse IgG (Vector Laboratories, Burlingame, CA, USA)
p53	Mouse monoclonal DO7 (# M7001)	Tris EDTA, pH 9.0, 125°C for 5 min; 20 min cool down	Dako Mouse Envision HRP System (Dako North America, Inc., Carpentaria, CA, USA)
MDM2	Mouse monoclonal OP46 (# OP46-100UG)	Tris Buffer pH 9.0, 125°C for 5 min; 20 min cool down	Dako Mouse Envision HRP System (Dako North America, Inc., Carpentaria, CA, USA)
RABL6A	Mouse monoclonal (8D1); 1:250 overnight at 4°C	HIER, Citrate buffer pH 6.0, 125°C for 5 min; 20 min cool down	Dako Mouse Envision HRP System (Dako North America, Inc., Carpentaria, CA, USA)
